# Anti-Aging Effects of Hydrophobic and Hydrophilic Components From Immature Pear Fruits Extract

**DOI:** 10.1093/geroni/igaa057.427

**Published:** 2020-12-16

**Authors:** Akira Ogita, Wakae Murata, Ken Yamauchi, Akiko Sakai, Yoshihiro Yamaguchi, Toshio Tanaka, Ken-ichi Fujita

**Affiliations:** 1 Osaka City University, Osaka, Japan; 2 Yonago National College of Technology, Yonago, Tottori, Japan; 3 Keio University, Yokohama, Kanagawa, Japan; 4 Osaka City University, Osaka, Osaka, Japan

## Abstract

Cellular senescence, the decline of cellular function due to aging, causes gradual loss of physiological functions and induces some chronic diseases, which negatively affect the quality of human life. Intervention in the cellular senescence process may reduce these incidences and delay the progression of age-related diseases, thereby contributing to the longevity of human lifespan. The budding yeast, Saccharomyces cerevisiae, is a model system that can provide significant insights into the genetics and molecular biology of senescence and is a suitable cellular model for research on mammalian cells. In the 2019 GSA meeting, we had revealed that the prolongation of yeast cell lifespan was induced by the addition of immature pear fruits extracts (iPE). In this study, we have focused on investigating the anti-senescence effects of hydrophilic (WiPE) and hydrophobic (OiPE) components of iPE on yeast cells and their genes and their possible application in extending human lifespan. The anti-aging effects of iPE were investigated using a chronological lifespan assay on S. cerevisiae cells. The chronological lifespan of the yeast was significantly extended in those treated with both WiPE and OiPE at 1% (v/v). The expression of sirtuin-related genes, which regulate cellular senescence, was examined by RT-PCR. Interestingly, gene expression was found to be significantly increased only in WiPE treated cells. The results suggested that the different polarity components from iPE exhibited anti-aging effects on the cells via different mechanisms. Research on the identification of useful components in iPE and the possibility of application to mammalian cells is ongoing.

